# N-linked glycosylation of NS1 protein modulates progeny virion assembly in orthoflaviviruses

**DOI:** 10.1371/journal.ppat.1014408

**Published:** 2026-07-09

**Authors:** Senzhao Zhang, Xuedan Tang, Zhen Wu, Hantai Tan, Yan Zhang, Wangyang Tan, Yu He, Tao Wang, Mingshu Wang, Renyong Jia, Dekang Zhu, Mafeng Liu, Xinxin Zhao, Qiao Yang, Ying Wu, Shaqiu Zhang, Juan Huang, Xumin Ou, Di Sun, Bin Tian, Anchun Cheng, Shun Chen

**Affiliations:** 1 Institute of Veterinary Medicine and Immunology, Sichuan Agricultural University, Chengdu, Sichuan, China; 2 Research Center of Avian Disease, College of Veterinary Medicine, Sichuan Agricultural University, Chengdu, Sichuan, China; 3 Agricultural Animal Diseases and Veterinary Public Health Key Laboratory of Sichuan Province, Sichuan Agricultural University, Chengdu, Sichuan, China; 4 Key Laboratory of Agricultural Bioinformatics, Ministry of Education, Sichuan Agricultural University, Chengdu, Sichuan, China; 5 Engineering Research Center of Southwest Animal Disease Prevention and Control Technology for Ministry of Education, Sichuan Agricultural University, Chengdu, Sichuan, China; 6 Institute of Veterinary Immunology and Green Drugs, Veterinary Department in College of Animal Science, State Key Laboratory of Green Pesticide, Guizhou University, Guiyang, China; Fundación Instituto Leloir-CONICET, ARGENTINA

## Abstract

Tembusu virus (TMUV) is a mosquito-borne avian virus belonging to the genus *Orthoflavivirus* within the family *Flaviviridae*. The nonstructural protein 1 (NS1) of TMUV is a secretory protein containing three N-linked glycosylation sites at residues N130, N175, and N207. Using a reverse genetics system and site-directed mutagenesis, we revealed that NS1 deglycosylation impairs the proliferation of recombinant TMUV (rTMUV) in multiple cell types. By performing subcellular fractionation assay, we observed that NS1 deglycosylation significantly impairs viral assembly. Besides, NS1 deglycosylation impairs the thermostability of NS1 dimers but not their formation. Tunicamycin treatment and enzyme-linked immunosorbent assays demonstrated that deglycosylated NS1 significantly induces the endoplasmic reticulum (ER) stress, which in turn reduces the secretion of NS1. Immunofluorescence and coimmunoprecipitation assays further demonstrated that deglycosylated NS1 is largely retained in the ER and enhances its interaction with the E protein. Retention using selective hooks (RUSH)-based live-cell imaging assay revealed that NS1 deglycosylation disrupts the trafficking of E protein from the ER to the Golgi apparatus. In addition, cycloheximide chase analysis showed that NS1 deglycosylation impairs its solubilization and then causes rapid degradation of NS1 and E protein by the host proteasomal pathway. Notably, the efficient viral assembly through NS1 glycosylation is a common feature among flaviviruses (West Nile virus and Yellow Fever virus). Our results consistently demonstrated that the glycosylation modification process of NS1 is highly synchronized with the maturation and assembly of viral particles during their trafficking from the ER to the Golgi apparatus. Collectively, our study confirms that NS1 glycosylation of *orthoflavivirus* species regulates progeny virion assembly by modulating the NS1-E interaction.

## Author summary

Tembusu virus (TMUV) is an avian orthoflavivirus and causing significant economic losses to poultry industry. The nonstructural protein 1 (NS1) of orthoflavivirus is multifunctional; however, the mechanism by which NS1 regulates flavivirus virion assembly remains largely unelucidated. Here, we demonstrated that NS1 deglycosylation leads to the retention of NS1 in the endoplasmic reticulum (ER). This retention prevents further and complex glycosylation of NS1 in the Golgi apparatus, thereby disrupting the NS1 secretory pathway and reducing NS1 secretion. Importantly, NS1 deglycosylation was found to inhibit the ER to Golgi trafficking of E proteins via the enhanced interaction between NS1 and E proteins. This in turn induces ER stress and subsequently triggers the rapid degradation of both retained NS1 and E proteins via the host proteasomal pathway. As a consequence, progeny virion assembly is obviously disrupted, and the production of infectious viral particles is largely reduced. Notably, the dependence of efficient progeny viral particle production on NS1 glycosylation represents a universal mechanism across several orthoflaviviruses. These findings unveil the molecular mechanism by which N-glycosylation of orthoflavivirus NS1 regulates the assembly of progeny virions.

## Introduction

The genus *Orthoflavivirus* (family *Flaviviridae*) comprises over 70 viruses, many of which, such as the dengue virus (DENV), Zika virus (ZIKV), yellow fever virus (YFV), West Nile virus (WNV), and Japanese encephalitis virus (JEV), are key human pathogens [1,2]. Tembusu virus (TMUV), a member of the genus *Orthoflavivirus*, has been identified as the causative agent of acute egg-drop syndrome in poultry [3]. Since its emergence in 2010, TMUV has rapidly spread across poultry populations in China and Southeast Asia (Malaysia and Thailand), causing substantial economic losses [4,5]. TMUV has a broad range of avian hosts (e.g., ducks, chickens, geese, pigeons, sparrows, and quails) [6,7] and replicates efficiently in various mammalian cell lines (e.g., BHK21, HeLa, A549, HepG2, SH-SY5Y and Vero) [8]. Moreover, anti-TMUV antibodies and viral RNA have been detected in serum samples and oral swabs of duck industry workers, respectively [9]. A recent study reported a natural TMUV infections in dolphins in Thailand [10], indicating its potential transmission from avian to mammalian hosts. Thus, the expanding host range and potential cross-species transmission of TMUV pose a great threat to global public health.

Similar to other orthoflavivirus members, enveloped TMUV carries a single-stranded positive-sense RNA genome with an open reading frame flanked by 5’ and 3’ untranslated regions. Cleavage of this polyprotein generates three structural proteins (capsid [C], premembrane [prM], and envelope [E]) and seven nonstructural proteins (NS1, NS2A, NS2B, NS3, NS4A, NS4B, and NS5) [11]. Highly conserved among orthoflaviviruses, NS1 is a multifunctional glycoprotein consisting of 352 amino acids, with a molecular weight (MW) ranging from 46-55 kDa depending on its N-glycosylation status [12]. After the addition of high-mannose-type glycans in the ER, NS1 is divided into three distinct populations: one portion interacts with other viral proteins to participate in viral replication [13–18]. Another portion of NS1 is trafficked to the plasma membrane via an unknown pathway, where it either associates with cholesterol/lipid rafts on the plasma membrane (mNS1) [19] or is modified with glycophosphatidylinositol (GPI), a modification that facilitates the anchoring of NS1 to the plasma membrane [20]. The remaining is trafficked to the Golgi apparatus for further processing, finally forming a soluble hexamer (sNS1) that is secreted into the extracellular environment [21]. sNS1 has been demonstrated to play a critical role in immune evasion [22–25], viral pathogenesis [26–28], and viral transmission [29], respectively.

N-glycosylation is a post-translational modification found in many secreted proteins, characterized by a consensus motif (N-X-S/T, where X is not proline) [30]. During translation, newly synthesized NS1 is translocated into the ER lumen via a signal peptide encoded in the C-terminal 24 amino acids of the E protein [31]. NS1 then undergoes initial N-glycosylation through the addition of high mannose-type glycans by the oligosaccharyl transferase complex within the ER [32]. Following this modification, glycosylated NS1 is transported to the Golgi apparatus, where the high mannose-type glycans are further trimmed by glycosidases and processed into complex-type glycans by glycosyltransferases in a stepwise manner [33]. Most members of the genus *orthoflavivirus* have two N-linked glycosylation sites in their NS1 at asparagine (N) residues 130 and 207, including all four DENV serotypes, JEV and ZIKV [34]; YFV NS1 has glycosylation sites at the N130 and N208 [35]. Several of these avian orthoflaviviruses—including TMUV, WNV, Usutu virus (USUV), Murray Valley encephalitis virus (MVEV) and Saint Louis encephalitis virus (SLEV), have an additional glycosylation site at N175 [36,37]. It is worth noting that for orthoflavivirus NS1, the glycans at residues N130 and N175 are complex-type, whereas the glycan at residue N207 is high-mannose-type.[33]. Compared to wild-type (WT) viruses, deglycosylated NS1 mutants of DENV, YFV and WNV exhibit decreased neurovirulence in mice [35,36,38,39], indicating that NS1 N-glycosylation is associated with viral pathogenicity. Mutations in NS1 N-glycosylation sites also decrease infectious particle production *in vitro* [40–42]. Our previously study consistently found that the deglycosylated NS1 mutant of TMUV exhibited impaired infectious particle production *in vitro* and reduced pathogenicity in ducklings [37]. Moreover, recent studies have further identified a novel role of NS1 in infectious particle production [43–45]. However, the role of NS1 N-glycosylation in regulating the assembly of progeny virions has not been elucidated.

In this study, to elucidate the underlying molecular mechanisms, we investigated the effect of NS1 N-glycosylation on virus assembly. Using reverse genetics and site-directed mutagenesis approaches, we constructed recombinant viruses and recombinant NS1, and found that NS1 deglycosylation blocks its secretory pathway from the ER to the Golgi apparatus, thereby reducing the secretion of NS1. Furthermore, we observed that deglycosylated NS1 exhibits enhanced interaction with the E protein, which in turn blocks E protein trafficking from the ER to the Golgi apparatus. In addition, NS1 deglycosylation impairs its solubilization and induces the ER stress, leading to degradation of both deglycosylated NS1 and E protein via the proteasomal pathway, ultimately resulting in attenuated assembly of progeny virions and reduced viral proliferation. Moreover, we observed consistent effects when extending our investigations to WNV_Kunjin_ and YFV, revealing that the dependence of efficient production of progeny viral particles on NS1 glycosylation is a conserved feature among orthoflaviviruses. Collectively, our study elucidates a novel mechanism by which NS1 N-glycosylation regulates assembly of progeny virions, thereby providing a better understanding of the assembly process of orthoflaviviruses from the ER to the Golgi apparatus.

## Materials and methods

### Cells, viruses, antibodies and plasmids

Duck embryo fibroblast (DEF) cells were isolated from nine-day-old duck embryos and maintained in Dulbecco’s modified Eagle’s medium (DMEM) (BasalMedia, Shanghai, China) containing 10% newborn calf serum (Gibco, Shanghai, China) and 1% penicillin/streptomycin (Solarbio, China). Chicken macrophage-like (HD11), african green monkey kidney (Vero), baby hamster kidney (BHK-21), stable TMUV-NS1-expressing BHK-21 (BHK-21-TMUV-NS1) and human cervical cancer (Hela) cells were cultured in DMEM supplemented with 10% fetal bovine serum (FBS) (Gibco, New York, USA) and 1% penicillin/streptomycin. Human embryonic kidney (HEK-293T) cells were cultured in RPMI 1640 basic medium (Gibco, Beijing, China) supplemented with 10% FBS and 1% penicillin/streptomycin. All cells were cultured at 37°C in a 5% CO_2_ atmosphere. The parental TMUV strain CQW1 (GenBank ID: KM233707.1) (rTMUV) was rescued from the full-length infectious clone of TMUV (pACNR FL-TMUV CQW1) and four recombinant viruses (rTMUV-NS1_N130A_, rTMUV-NS1_N175A_, rTMUV-NS1_N207A_ and rTMUV-NS1_N130A/N175A/N207A_) were provided by our laboratory as previously reported [46]. The parental WNV_kunjin_ strain FLSDX (GenBank ID: AY274504.1) (rWNV_kunjin_) and parental YFV strain 17D (GenBank ID: MT107250.1) (rYFV) were rescued from two full-length infectious clone (pUC19 FL-WNV_Kunjin_ and PACYC FL-YFV) provided by our laboratory; their recombinant mutants were constructed using the pUC19 FL-WNV_Kunjin_ or PACYC FL-YFV as backbones [47]. Virus stocks were propagated in BHK-21 cells, aliquoted, and stored at −80°C. Anti-TMUV E polyclonal antibodies (pAbs), anti-TMUV NS1 monoclonal antibodies (mAbs) (clone 8E6, 10F12 and 3D5), and anti-TMUV E mAbs were provided by our laboratory as previously [48]. Mouse anti-Flag tag mAb was purchased from MBL Biotech (Beijing, China). Anti-GAPDH, anti-His tag, anti-β-tubulin, anti-GM130, anti-GFP tag, anti-PERK, anti-p-PERK mAbs and anti-GRP78 pAbs, were purchased from ABclonal (Wuhan, China). Anti-XBP1s and anti-ATF6 mAbs were purchased from Cell Signaling Technology. Rabbit anti-strep tag mAb was purchased from HUABIO (Hangzhou, China). Anti-calnexin and anti-HA tag mabs were purchased from Proteintech (Wuhan, China). Alexa Fluor 488-conjugated anti-mouse IgG and Alexa Fluor 568-conjugated anti-rabbit IgG were purchased from Thermo Fisher Scientific (Shanghai, China). pDsRed2-ER and pDsRed2-Golgi were provided by our laboratory [37]. pLV2-Str-HA_Tag_-Li-IRES-VSV G-SBP-GFP was kindly donated by Professor Hongbo Zhou (Huazhong Agricultural University, Wuhan, China) [49]. All expression plasmids used in this study are summarized in Table 1.

**Table 1:** Plasmid list.

Plasmid name	Template source	Backbone	Cloning Sites
pcDNA3.1-NS1–2*Strep	pACYC-TMUV-Nluc-rep	pcDNA3.1-2*Strep	EcoRI/NotI
pcDNA3.1-E-2*Strep	pACNR FL-TMUV CQW1	pcDNA3.1-2*Strep	EcoRI/NotI
pCAGGS-NS1-HiBiT	pACYC-TMUV-Nluc-rep	pCAGGS	EcoRI/BglII
pCAGGS-NS1_N130A_-HiBiT	pACYC-TMUV-Nluc-rep	pCAGGS	EcoRI/BglII
pCAGGS-NS1_N175A_-HiBiT	pACYC-TMUV-Nluc-rep	pCAGGS	EcoRI/BglII
pCAGGS-NS1_N207A_-HiBiT	pACYC-TMUV-Nluc-rep	pCAGGS	EcoRI/BglII
pCAGGS-NS1_N130A/N175A/N207A_-HiBiT	pACYC-TMUV-Nluc-rep	pCAGGS	EcoRI/BglII
pcDNA3.1-WNV_Kunjin_-E-GFP	pUC19 FL-WNV_Kunjin_	pcDNA3.1-GFP	EcoRI/XbaI
pcDNA3.1-WNV_Kunjin_-E-2*Strep	pUC19 FL-WNV_Kunjin_	pcDNA3.1-2*Strep	EcoRI/NotI
pCAGGS-WNV_Kunjin_-NS1-Flag	pACYC-WNV_Kunjin_-Nluc-rep	pCAGGS	EcoRI/BglII
pCAGGS-WNV_Kunjin_-NS1_N130A/N175A/N207A_-Flag	pACYC-WNV_Kunjin_-Nluc-rep	pCAGGS	EcoRI/BglII
pcDNA3.1-YFV-E-GFP	PACYC FL-YFV	pcDNA3.1-GFP	EcoRI/XbaI
pcDNA3.1-YFV-E-2*Strep	PACYC FL-YFV	pcDNA3.1-2*Strep	EcoRI/NotI
pCAGGS-YFV-NS1-Flag	pACYC-YFV-Nluc-rep	pCAGGS	EcoRI/BglII
pCAGGS-YFV-NS1_N130A/N208A_-Flag	pACYC-YFV-Nluc-rep	pCAGGS	EcoRI/BglII
pLV2-Str-HA_Tag_-Li-IRES-E-SBP-GFP	pLV2-Str-HA_Tag_-Li-IRES-VSV G-SBP-GFP	pLV2-CMV-IRES-GFP	BamHI/SbfI

### Virus titration and growth kinetics

Viral titers were determined by the median tissue culture infectious dose 50 (TCID_50_) assays on BHK-21 cells as previously reported [46]. Serial 10-fold dilutions of viral samples were prepared by mixing 100 μl of each viral sample with 900 µl DMEM, 100 µL of each dilution was then added to each of 8 wells of a 96-well plate seeded with a monolayer of BHK-21 cells. Following incubation at 37°C with 5% CO₂ for 5–7 days, cytopathic effects were examined via microscopy, and viral titers were calculated using the Karber method. Growth kinetics of rTMUV and its mutants were compared in DEF, HD11, Vero, HEK-293T, BHK-21 and BHK-21-TMUV NS1 cells. Growth kinetics of the rWNV_Kunjin_, rYFV and their mutants were compared in BHK-21 cells. Subconfluent cell monolayers in 12-well plates were infected with rTMUV or various mutants at a dose of 300 TCID_50_. After 2 h incubation at 37°C with 5% CO_2_, the inoculum was removed, and cells were washed three times with phosphate-buffered saline (PBS). Cell culture medium supplemented with 2% FBS and 1% penicillin/streptomycin was then added to each plate. Supernatant were collected at indicated time points and subjected to viral titration as described above.

### Virus attachment, internalization and assembly efficiency assay

Subconfluent BHK-21 cell monolayers in 12-well plates were infected with rTMUV or recombinant viruses at an equal dose for 1 h at 4°C. For the virus attachment assay, unbound virus was removed by washing with PBS three times, then total RNA was extracted from cells to quantify viral RNA. For the virus internalization assay, unbound virus was removed by washing with PBS three times, and cells were maintained in DMEM containing 2% FBS for 1 h at 37°C, then total RNA was extracted from cells to quantify viral RNA. For the virus assembly efficiency assay, unbound virus was removed by washing with PBS three times, and cells were maintained in DMEM containing 2% FBS for 16 h at 37°C to ensure a single round of viral infection. Total RNA was then extracted from cells to quantify viral RNA, and cell culture supernatants were subjected to viral titration. Assembly efficiency was shown as relative infectivity (extracellular titer/intracellular viral RNA copies).

### RNA extraction and quantitative reverse transcription PCR (RT-qPCR)

Total cellular RNA was isolated using RNAiso Plus reagent (Takara, Dalian, China) and cDNA was generated using HiScript III RT SuperMix (Vazyme, Nanjing, China). TMUV genome copies were determined by quantitative PCR (qPCR) using F488 SYBR qPCR Mix (BestEnzymes Biotech Co., Ltd. Lianyungang, China) following standard cycling conditions as previously established by our laboratory [50].

### Subcellular fractionation

Subcellular fractionation was performed as previously described with minor modifications [51]. Subconfluent BHK-21 cell monolayers in 100-mm culture dishes were infected with rTMUV or rTMUV-NS1_N130A/N175A/N207A_ at an equal dose. Cells were trypsinized and washed three times with PBS. Cell pellets were resuspended in 1 ml hypo-osmotic buffer (10 mM HEPES-NaOH, pH 7.8) and allowed to swell on ice for 10 min, followed by centrifugation at 800 *g* for 2 min at 4°C. The medium was returned to iso-osmoticity by removal of 650 µl of the supernatant and the addition of 350 µl of hyperosmotic buffer (0.6 M sucrose, 10 mM HEPES-NaOH, pH 7.8). Cells were disrupted by being passed 50 times through a 25-G needle, and nuclei were removed via centrifugation at 12,000 rpm for 30 min at 4°C. The post-nuclear fraction was mixed with 700 µl of 60% iodixanol to prepare a 30% iodixanol solution. 10 and 20% iodixanol solutions were prepared by mixing with hypo-osmotic buffer. A total of 4.2 ml of these three solutions was layered into centrifuge tubes, which were centrifuged at 50,000 rpm in an MLS-50 rotor (Beckman) for 3 h at 4°C. Ten 420 µl fractions were collected from top to bottom. 200 µl of each fraction was used for RNA extraction and qPCR, 100 µl for western blot analysis, and the remaining 100 µl for viral titration by TCID_50_ assays. Calnexin, an endoplasmic reticulum-resident protein, served as a marker for western blot analysis.

### Western blot analysis

Cells were harvested at the indicated time points in RIPA buffer (Thermo Fisher Scientific, Shanghai, China) containing with 1 × protease inhibitor cocktail (MedChemExpress, Shanghai, China). Cell lysates were clarified by centrifugation at 12,000 rpm for 15 min at 4°C. Supernatants were either heated at 100°C for 10 min or left unheated (to detect NS1 dimers), then separated by 10% or 12% sodium dodecyl sulfate-polyacrylamide gel electrophoresis (SDS-PAGE) and subsequently transferred to PVDF membranes. Membranes were washed three times by TBST (TBS containing 0.1% Tween 20), blocked with 5% non-fat milk in TBST, and incubated with the indicated primary antibodies overnight at 4°C. Following three washes with TBST, membranes were incubated with horseradish peroxidase (HRP)-conjugated goat anti-mouse or goat anti-rabbit IgG secondary antibodies (Bio-Rad, USA) at a 1:3000 dilution for 1–2 h at room temperature. Protein signals were visualized using Clarity Western ECL Substrate (Bio-Rad, USA), and band intensities were quantified using ImageJ software.

### Coimmunoprecipitation (Co-IP) assay

HEK-293T cells were seeded in 60-mm culture dishes and incubated to 70–80% confluence. The indicated expression plasmids (a total amount of 4 μg) were co-transfected into subconfluent cell monolayers using Lipofectamine 3000 (Thermo Fisher Scientific, Shanghai, China). At 24 h post-transfection, cells were harvested in Pierce IP Lysis Buffer (Thermo Fisher Scientific, Shanghai, China) containing 1 × protease inhibitor cocktail, incubated at 4°C for 1 h, and cell lysates were clarified by centrifugation at 12,000 rpm for 15 min at 4°C. For each sample, the cell lysate was incubated with the indicated antibody overnight at 4°C, followed by incubation with 30 μl Protein A/G Magnetic Beads (MedChemExpress, Shanghai, China) for 4 h at 4°C. The beads were washed three times with 1 ml cold TBST. Finally, the precipitates were separated by SDS-PAGE, and western blot analysis was performed as described above.

### Thermostability assay

HEK-293T cells were seeded in 6-well plates to 70–80% confluence. The indicated recombinant NS1 expression plasmid (a total amount of 2 μg) was transfected into subconfluent cell monolayers using Lipofectamine 3000. At 36 h post-transfection, cells were harvested in Pierce IP Lysis Buffer as described above. Samples were incubated at 4°C, 37°C or 50°C for indicated times before being subjected to western blot analysis.

### Indirect enzyme-linked immunosorbent assay (ELISA)

Indirect ELISA was performed as previously established by our laboratory with some modifications [48]. Briefly, NS1 in culture supernatants from transfected or TMUV-infected cells was collected at the indicated time points. 96-well microtiter plates were coated with equal volumes of samples in 0.1 M carbonate buffer (pH 9.6) at 4°C overnight. Nonspecific binding sites in each well were blocked with 5% BSA in PBS at 37°C for 2 h. Plates were incubated with anti-NS1 mAb (clone 8E6) at 37°C for 1.5 h, followed by HRP-conjugated goat anti-mouse IgG at a 1:3000 dilution (Bio-Rad, USA) at 37°C for 1 h. Signals were developed with TMB (TIANGEN, Beijing, China) at room temperature for 15 min, and the reaction was terminated by adding 2 M H_2_SO_4_ before reading on a 96-well plate reader (Bio-Rad, USA) at 450 nm. Three washes with 0.1% Tween 20 in PBS (PBST) were performed between steps.

### Cell viability assay

The Cell Counting Kit-8 (CCK-8) (MedChemExpress, Shanghai, China) was used to evaluate the viability of BHK-21 cells when exposed to tunicamycin (Tun) (MedChemExpress, Shanghai, China), following the instructions provided by the manufacturer. Cells were seeded in 96-well plates with different concentrations of Tun (0, 0.625, 1.25, 2.5, 5, 10, 20, 40, 80 μg/ml) for 6 h. Following incubation with 10 μl CCK-8 solution for 1 h at 37°C, the OD_450_ value was detected with a microplate reader (Bio-Rad, USA). The relative cell viability was calculated with formula: A-C/B-C × 100%, where A = absorbance of experimental group (Tun treated cells), B = absorbance of control group (untreated cells), C = absorbance of blank group (without cells).

### Tunicamycin treatment

The indicated NS1 expression plasmid (a total amount of 2 μg) was transfected into subconfluent BHK-21 cell monolayers in 6-well plates. At 42 h post-transfection, cells were treated with 0.1% DMSO, 5 μg/ml or 10 μg/ml tunicamycin for 6 h. NS1 levels in supernatants and cell lysates were measured by western blot analysis. Additionally, subconfluent BHK-21 cell monolayers in 12-well plates were infected with rTMUV at 300 TCID_50_. At 42 h post-infection, cells were treated with 0.1% DMSO or 10 μg/ml tunicamycin for 6 h. NS1 levels in supernatants were quantified via indirect ELISA, and extracellular viral titers were measured.

### Deglycosylation assay

The deglycosylation assay was performed as described in our previous study [37]. Samples were treated with Endo H or PNGase F (New England BioLabs, Beijing, China) at 37˚C for 1 h. Then the reaction was subjected to western blot analysis.

### Immunofluorescence assay (IFA)

For co-localization analysis, cells were washed three times with cold PBS and fixed with 4% paraformaldehyde overnight at 4°C. Fixed cells were permeabilized with 0.3% Triton X-100 for 1 h at 4°C, then blocked with 5% bovine serum albumin (BSA) (Solarbio, China) for 1 h at 37°C. Cells were incubated with the indicated primary antibody at a 1:200 dilution overnight at 4°C, followed by incubation with Alexa Fluor 488-conjugated anti-mouse IgG (Thermo Fisher Scientific, Shanghai, China) as the secondary antibody at a 1:1000 dilution for 2 h at 37°C. Finally, cells were counterstained with 4’, 6-diamidino-2-phenylindole (DAPI) (Solarbio, China) for 15 min at room temperature. Between each step, cells were washed three times with cold PBST. Slides were imaged using an FV3000 confocal laser scanning microscope (Olympus).

### HiBiT activity detection

Subconfluent BHK-21 cell monolayers in 100-mm culture dishes were transfected with the indicated expression plasmid. After 48 h post-transfection, cells were subjected to subcellular fractionation. The HiBiT tag in each fraction was detected by a Nano-Glo HiBiT Lytic Detection System (Promega, USA) and a GloMax Navigator System (Promega, USA), following the manufacturer’s protocol.

### Retention using selective hooks (RUSH) assays

For the RUSH system, the reporter gene (VSV G) was replaced with TMUV E and cloned into the linearized pLV2-Str-HA tag-Li-IRES-VSV G-SBP-GFP vector (linearized using *Bam*HI and *Sbf*I restriction enzymes). BHK-21 cells in 35-mm confocal dishes (Cellvis) were co-transfected with pLV2-Str-HA_Tag_-Li-IRES-E-SBP-GFP, pDsRed2-ER and indicated NS1-expression plasmids for 24 h. To activate the RUSH system, biotin (400 µM; Sigma, B4639) was added to each dish to release cargo proteins anchored on the ER. Cargo transport was visualized via Cell Xpanse (CSR Biotech, Guangzhou, China) at indicated time points. The representative viral membrane protein, VSV G, was demonstrated to transport from the ER to the Golgi apparatus. Thus, the pLV2-Str-HA_Tag_-Li-IRES-VSV G-SBP-GFP was used as a positive control. For co-localization analysis, BHK-21 cells were seeded on coverslips placed in 12-well plates and allowed to adhere. The cells were then co-transfected with pLV2-Str-HA_Tag_-Li-IRES-E-SBP-GFP and indicated NS1-expression plasmids for 24 h, followed by the addition of biotin (400 µM) to the culture medium for 30 min. Cells were then fixed and stained with indicated antibodies, and finally visualized via FV3000 confocal microscopy using IFA. The co-localization of E protein with the ER or the Golgi apparatus was analyzed using Fiji software.

### Solubility detection of NS1

Cells were harvested at the indicated time points in RIPA Buffer containing with 1 × protease inhibitor cocktail, and the solubility of NS1 was compared between wild-type and mutant NS1. After centrifugation at 12,000 rpm for 15 min at 4°C, the supernatant (soluble fraction) and pellets (insoluble fraction) were analyzed to determine NS1 levels via western blot analysis or HiBiT activity detection.

### Cycloheximide (CHX) chase analysis

To assess degradation of NS1, cells were treated with 50 µg/ml CHX and 10 µM MG132 at 24 h post-transfection, then harvested at indicated time points. Cell lysates were subjected to western blot analysis. Band intensities were quantified using ImageJ software.

### Plaque assay

The plaque assay was performed as previously described with minor modifications [37]. Subconfluent BHK-21 cell monolayers in 12-well plates were infected with viral samples at a dose of 50 TCID_50_ and incubated at 37°C for 1 h. Cells were washed three times with PBS, followed by overlay with DMEM containing 2% FBS and 1% methyl cellulose. After incubation at 37°C with 5% CO_2_ for 6 days, cells were fixed with 4% formaldehyde for 20 min and stained with 1% crystal violet solution for 1 min. Plaque sizes were measured using ImageJ software.

### Quantifcation and statistical analysis

All data were derived from three independent experiments and analyzed using GraphPad Prism 9.5 software. Differences were considered statistically significant when P < 0.05. Statistical significance is indicated as follows: *, P < 0.05; **, P < 0.01; ***, P < 0.001.

## Results

### Deglycosylation of TMUV NS1 impairs viral assembly

**Fig 1:**
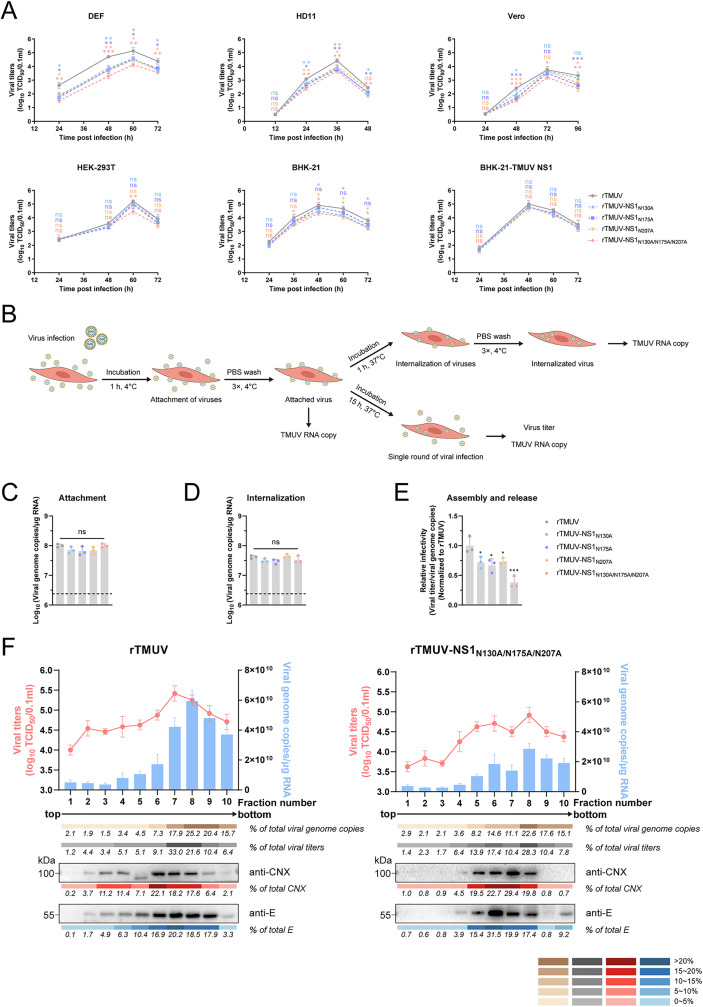
Deglycosylation of TMUV NS1 impairs viral assembly. **(A)** Growth kinetics of rTMUV and its mutants in different cell types. DEF, HD11, Vero, HEK-293T, BHK-21, or BHK-21-TMUV-NS1 cells in 12-well plates were infected with rTMUV or various mutants at 300 TCID_50_, and the culture supernatants were collected at the indicated time points. The viral titers were then determined by TCID_50_ assays. Means and SDs from three independent experiments are presented. *, P < 0.05; **, P < 0.01; ***, P < 0.001; ns, not significant. **(B)** Schematic diagram for the virus attachment, internalization and assembly efficiency assay. **(C and D)** BHK-21 cells in 12-well plates were infected with equal dose of rTMUV or various mutants for 1 h at 4°C. **(C)** For attachment assay, unbound virus was removed by washing with PBS three times, then total RNA was extracted from cells to quantify viral RNA by qPCR. **(D)** For the virus internalization assay, unbound virus was removed by washing with PBS three times, and cells were kept for 1 h at 37°C to ensure the internalization of virus, then total RNA was extracted from cells to quantify viral RNA by qPCR. **(E)** For the virus assembly efficiency assay, BHK-21 cells in 12-well plates were infected with equal dose of rTMUV or various mutants for 1 h at 37°C, then cells were washed with PBS three times and were kept for 16 h at 37°C to ensure single round of viral infection, then total RNA was extracted from cells to quantify viral RNA by qPCR and cell culture supernatant was subjected to viral titration by TCID_50_ assays. The assembly efficiency was shown as relative infectivity (extracellular viral titer/intracellular viral genome copies) and normalized to rTMUV. **(F)** Subcellular fractionation of infected BHK-21 cells. BHK-21 cells in 100-mm culture dishes were infected with equal dose of rTMUV or rTMUV-NS1_N130A/N175A/N207A_.The cellular contents were analyzed by fractionation on an iodixanol gradient. Ten fractions were collected from the top of the gradient, and the viral titer of each fraction was quantified by TCID_50_ assay. The viral genome copies contained in each fraction was quantified by qPCR. Means and SDs from three independent experiments are presented. Each fraction was also evaluated by western blot analysis for the presence of E protein using calnexin (CNX) as a marker. The intensities of the indicated protein bands were quantified by using ImageJ software. Data were shown as the E in each fraction/total E and CNX in each fraction/total CNX.

To examine the effect of NS1 glycosylation mutations on TMUV growth, we produced rTMUV and recombinant viruses and evaluated their proliferation in avian duck embryo fibroblast DEF and chicken macrophage-like HD11 cells firstly. We could detect a notable reduction of viral titers of all recombinant viruses compared to that of rTMUV, and the peak titer of rTMUV-NS1_N130A/N175A/N207A_ was significantly impaired around 9-fold and 4.6-fold in DEF and HD11 cells, respectively (Fig 1A). We consistently found that the peak titer of rTMUV-NS1_N130A/N175A/N207A_ reduced around 2.5-fold, 4.6-fold and 2.8-fold in mammalian Vero, HEK-293T and BHK-21 cells respectively, compared to that of rTMUV (Fig 1A). In addition, we used stable NS1-expressing BHK-21 cells and found that rTMUV-NS1_N130A/N175A/N207A_ could recover its proliferation by homologous helper NS1 supplied in *trans* (Fig 1A), indicating that exogenous NS1 is functional and can be utilized for a series of *in vitro* assays. We further explored the impact of NS1 deglycosylation on the viral life cycle, including attachment, internalization, assembly and release (Fig 1B). Results clearly demonstrated that NS1 deglycosylation did not impair viral attachment (Fig 1C) or viral internalization (Fig 1D). Interestingly, NS1 deglycosylation impairs the viral assembly and release (Fig 1E), with the viral assembly/release efficiency of the mutants reduced by 28%, 33%, 27%, and 62%, respectively. To further characterize the effect of NS1 deglycosylation on the assembly of progeny virions, we fractionated cell lysates via gradient centrifugation and collected ten fractions from top to bottom. We then analyzed the viral titer, viral genome copies, and E protein level in each fraction. Since orthoflavivirus assembly sites are associated with endoplasmic reticulum (ER) [52], we used an ER resident protein calnexin (CNX), as a marker. Most of the CNX, E protein, viral titer and viral genome copies generally accumulated in fractions 7 and 8 for the WT virus, reflecting proper viral assembly at the ER (Fig 1F, left panel). For the mutant virus, the virion assembly indicators exhibited two key changes: the viral titer and viral genome copies were significantly reduced (with two peaks in fractions 6 and 8); the viral titer and viral genome copies peak in fraction 8, whereas the E protein is peaks in fraction 6, indicating impaired virion assembly (Fig 1F, right panel). Consistent with previous studies, this shift may result from the inability of viral particles to enter the secretory pathway [53,54]. Taken together, these results demonstrate that NS1 deglycosylation impairs viral assembly.

### Glycosylation is essential for the thermal stability of NS1 dimer and the secretion of NS1

**Fig 2:**
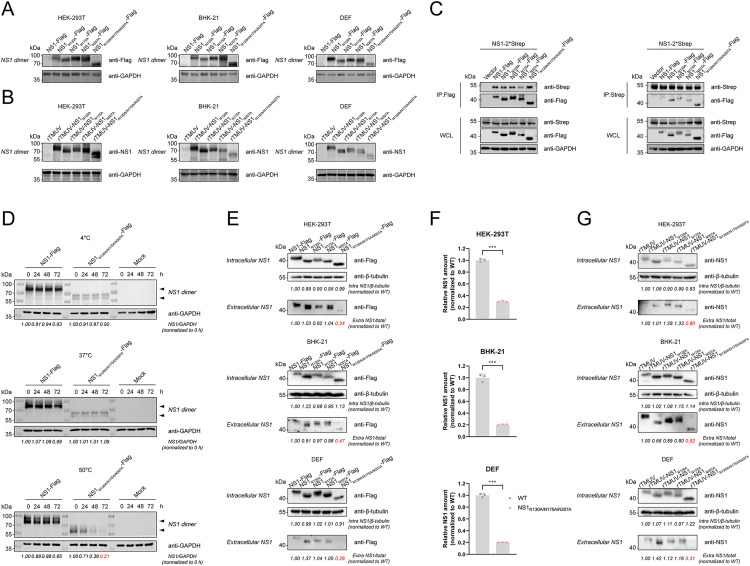
Glycosylation is essential for NS1 dimer thermostability and NS1 secretion. (A) Detection of NS1 dimer in transfected HEK-293T, BHK-21 or DEF cells. Cells in 6-well plates were transfected with the indicated NS1-expression plasmids (2000 ng/well) for 36 h. Samples were without heat treatment and subjected to western blot analysis using anti-Flag mAb (1:5000) or anti-GAPDH mAb (1:5000). (B) Detection of NS1 dimer in TMUV-infected HEK-293T, BHK-21 or DEF cells. Cells in 6-well plates were infected with the rTMUV or various mutants at 3000 TCID_50_. Cell lysates were harvested at 48 h post infection without heat treatment and subjected to western blot analysis using anti-NS1 mAb (clone 10F12) or anti-GAPDH mAb (1:5000). (C) Detection the interaction between NS1 itself. HEK-293T cells in 60-mm culture dishes were co-transfected with NS1–2*Strep (2000 ng/dish) and the indicated NS1-expression plasmids (2000 ng/dish) for 24 h. The cell lysate then was subjected to coimmunoprecipitation and western blot analysis using anti-Flag mAb (1:5000), anti-Strep mAb (1:3000) or anti-GAPDH mAb (1:5000). (D) Thermostability analysis of NS1 dimers. HEK-293T cells in 6-well plates were transfected with the indicated NS1-expression plasmids (2000 ng/well) for 36 h. The cell lysate was exposed to 4°C, 37°C or 50°C for indicated time and then subjected to western blot analysis using anti-Flag mAb (1:5000) or anti-GAPDH mAb (1:5000). The intensities of the indicated protein bands were quantified by using ImageJ software. Data were shown as the fold-change of NS1/GAPDH. Filled triangles correspond to NS1 dimers. (E) Detection of intracellular and extracellular NS1 in transfected cells. HEK-293T, BHK-21 or DEF cells in 6-well plates were transfected with the indicated NS1-expression plasmids (2000 ng/well). Cell culture supernatant and cell lysates were harvested at 48 h post transfection and subjected to western blot analysis using anti-Flag mAb (1:5000) or anti-β-tubulin mAb (1:5000). The secretion rate of NS1 was quantified using the following formula: extracellular NS1/ (extracellular NS1 + intracellular NS1), where extracellular NS1 = NS1 in cell culture supernatant, intracellular NS1 = NS1 in cell lysate. The intensities of the indicated protein bands were quantified by using ImageJ software. Data were shown as the fold-change of intracellular NS1/β-tubulin or extracellular NS1/total NS1. (F) Detection of levels of NS1 in supernatants. Supernatants were collected from (E), and NS1 levels in HEK-293T, BHK-21 or DEF cells were quantified by indirect ELISA using anti-NS1 mAb (clone 8E6). The relative NS1 amount was normalized to WT group. Means and SDs from three independent experiments are presented. ***, P < 0.001. (G) Detection of intracellular and extracellular NS1 in TMUV-infected cells. HEK-293T, BHK-21 or DEF cells in 12-well plates were infected with the rTMUV or various mutants at 300 TCID_50_. Cell culture supernatant and cell lysate was harvested at 48 h post infection and subjected to western blot analysis using anti-NS1 mAb (clone 10F12) or anti-β-tubulin mAb (1:5000). Data were shown as described in (E).

We next investigated the effect of NS1 deglycosylation on the biological properties of NS1. Consistent with previous studies [55,56], NS1 dimers were detectable in HEK-293T, BHK-21, and DEF cells following either overexpression (Fig 2A) or viral infection (Fig 2B), indicating that glycosylation of NS1 is dispensable for its dimerization. Coimmunoprecipitation (Co-IP) results further revealed that deglycosylation does not disrupt interactions between NS1 itself (Fig 2C). Given that glycosylation is known to be associated with the proper folding and stability of glycoproteins [57,58], we further assessed NS1 thermostability by exposing protein samples to different temperatures before being subjected to western blot analysis. We confirmed that all recombinant NS1 dimers remained stable at 4°C and 37°C (Fig 2D, upper and middle panels), whereas the NS1_N130A/N175A/N207A_ dimer exhibited the highest heat sensitivity, with a gradual reduction in protein levels over time at 50°C (Fig 2D, bottom panel). Since glycosylation is also essential for NS1 secretion [59,60], supernatants and cell lysates were collected from plasmid-transfected cells at 48 h post transfection and analyzed via western blot (Fig 2E) and indirect ELISA (Fig 2F). Notably, no significant difference was observed in intracellular NS1 levels between WT and glycosylation mutants; however, the secretion rate of NS1_N130A/N175A/N207A_ was reduced by approximately 76%, 53%, and 72% in HEK-293T, BHK-21, and DEF cells, respectively (Fig 2E). Relative amount of NS1_N130A/N175A/N207A_ exhibited 70.1%, 79.6% and 79.9% reduction in HEK-293T, BHK-21 and DEF cells, respectively (Fig 2F). Consistently, the secretion rate of NS1_N130A/N175A/N207A_ was also reduced by 20%, 48% and 69% in HEK-293T, BHK-21 and DEF cells during TMUV infection, respectively (Fig 2G). Taken together, these findings indicate that deglycosylation does not impair NS1 dimer formation, but is critical for the thermostability of NS1 dimers and NS1 secretion.

### NS1 deglycosylation induces ER stress, which leads to reduced NS1 secretion

**Fig 3:**
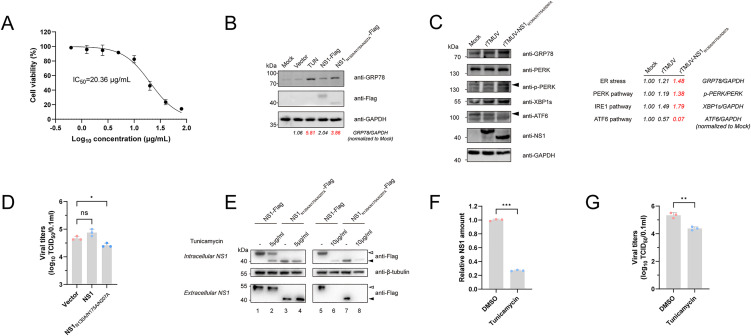
NS1 deglycosylation induces ER stress, leading to reduced NS1 secretion. **(A)** Detection of cell viability. CCK-8 assay showing the viability of BHK-21 cells after treatment with different concentrations of tunicamycin (0, 0.625, 1.25, 2.5, 5, 10, 20, 40, 80 μg/ml) for 6 h in BHK-21 cells. Means and SDs from three independent experiments are presented. **(B)** Effects of NS1 deglycosylation on ER stress. BHK-21 cells in 12-well plates were transfected with the indicated NS1-expression plasmids (1000 ng/well). Cell lysate was harvested at 48 h post transfection for western blot analysis using anti-GRP78 pAb (1:3000) or anti-GAPDH mAb (1:5000). Cells treated with 10 mg/ml tunicamycin for 6 h were served as a positive control. The intensities of the indicated protein bands were quantified by using ImageJ software. Data were shown as the fold-change of GRP78/GAPDH. **(C)** Effects of NS1 deglycosylation on the three UPR signaling branches. Hela cells in 6-well plates were infected with the rTMUV or rTMUV-NS1_N130A/N175A/N207A_ at 10^4^ TCID_50_. Cell lysates were harvested at 48 h post infection and subjected to western blot analysis using anti-GRP78 pAb (1:3000), anti-PERK mAb (1:1000), anti-p-PERK mAb (1:1000), anti-XBP1s mAb (1:1000), anti-ATF6 mAb (1:1000), anti-NS1 mAb (clone 10F12) or anti-GAPDH mAb (1:5000). The intensities of the indicated protein bands were quantified by using ImageJ software. Data were shown as the fold-change of GRP78/GAPDH, p-PERK/PERK, XBP1s/GAPDH and ATF6/GAPDH, respectively. **(D)** Detection of viral titers after overexpression with NS1 or NS1_N130A/N175A/N207A_. BHK-21 cells in 12-well plates were transfected with the indicated NS1-expression plasmids (1000 ng/well). At 12 h post transfection, cells were infected with rTMUV at 300 TCID_50_. Cell culture supernatants were collected at 48 h post infection and viral titers were then determined by TCID_50_ assays. Means and SDs from three independent experiments are presented. *, P < 0.05; ns, not significant. **(E)** Detection of intracellular and extracellular NS1 after treatment with tunicamycin. BHK-21 cells in 6-well plates were transfected with the indicated NS1-expression plasmids (2000 ng/well). At 42 h post transfection, cells were treated with 5 mg/ml or 10 mg/ml tunicamycin in 0.1% DMSO for 6 **h.** Cell culture supernatant and cell lysate was harvested and subjected to western blot analysis using anti-Flag mAb (1:5000) or anti-β-tubulin mAb (1:5000). Empty triangles represent glycosylated NS1, while filled triangles correspond to unglycosylated NS1. **(F and G)** Detection of levels of NS1 and viral titers in supernatants from TMUV-infected cells after treatment with tunicamycin. BHK-21 cells in 12-well plates were infected with the rTMUV at 300 TCID_50_. At 42 h post infection, cells were treated with either 0.1% DMSO or 10 mg/ml tunicamycin in 0.1% DMSO for 6 **h.** Cell culture supernatant was harvested for indirect ELISA (F) or TCID_50_ assays **(G)**. Means and SDs from three independent experiments are presented. **, P < 0.01; ***, P < 0.001.

Since NS1 is a secreted protein, ER retention of NS1 would impair its subsequent secretion. We hypothesized that ER-retained NS1 not only impairs its own secretion but also induces ER stress. ER stress arises from the accumulation of unfolded or misfolded proteins in the ER, which triggers the unfolded protein response (UPR) to sustain cellular homeostasis [61]. The UPR encompasses three signaling branches: the RNA-activated protein kinase-like endoplasmic reticulum kinase (PERK) pathway, the inositol-requiring enzyme 1 (IRE1) pathway, and the activating transcription factor 6 (ATF6) pathway [61]. Under resting conditions, glucose-regulated protein 78 (GRP78) binds to these ER-resident sensors; upon ER stress, GRP78 dissociates from these sensors, leading to UPR activation [62]. Thus, upregulated GRP78 expression serves as a core molecular marker of ER stress. During ER stress, PERK is activated through autophosphorylation and subsequently phosphorylates eukaryotic initiation factor 2α (eIF2α). IRE1 also undergoes autophosphorylation, which enables it to mediate the splicing of unspliced X-box binding protein 1 (XBP1u) into spliced XBP1 (XBP1s). In parallel, ATF6 is cleaved to produce the N-terminal fragment ATF6-N[62]. Thus, we used tunicamycin (TUN), which inhibits N-glycosylation and causes accumulation of unfolded proteins in the ER [62,63], as an ER stress agonist. Cell viability was assessed using the cell counting kit-8 (CCK-8) assay. Results showed that BHK-21 cells retained viability at a TUN concentration of 10 μg/ml (Fig 3A). Next, we examined whether NS1 deglycosylation induces ER stress. Notably, overexpression of NS1_N130A/N175A/N207A_ significantly upregulated GRP78 levels (Fig 3B). We further determined whether deglycosylation activates the three branches of UPR under TMUV infection. Consistent with previous study by our colleagues [62], infection with WT virus led to the increased expression of p-PERK and XBP1s whereas the reduced expression of full-length ATF6, indicating that the activation of all three pathways; moreover, the mutant virus induced stronger activation compared with the WT virus (Fig 3C). Overexpression of NS1_N130A/N175A/N207A_ also reduced infectivity titer compared to those of control group (Fig 3D). We then investigated the effect of ER stress on intracellular and extracellular NS1 expression, as well as on viral particle formation. With increasing TUN concentrations, the intracellular and extracellular expression levels of both WT NS1 and NS1_N130A/N175A/N207A_ were significantly reduced (empty triangle represents WT NS1 and filled triangle corresponds to the deglycosylated NS1) (Fig 3E, lane 2 vs. 6 and lane 4 vs. 8). We consistently observed that the relative amount of NS1 (Fig 3F) and viral titers (Fig 3G) in the culture supernatants of TMUV-infected cells were significantly reduced following treatment with 10 μg/ml TUN. Given that both NS1 and the E protein are viral glycoproteins, the observed reduction in infectious virion production upon tunicamycin treatment can be reasonably ascribed to the synergistic effects of impaired glycosylation of both proteins. Collectively, our data demonstrate that NS1 deglycosylation induces ER stress, which reduces the level of extracellular NS1 from TMUV-infected cells.

### Transport of deglycosylated NS1 is arrested at the ER

**Fig 4:**
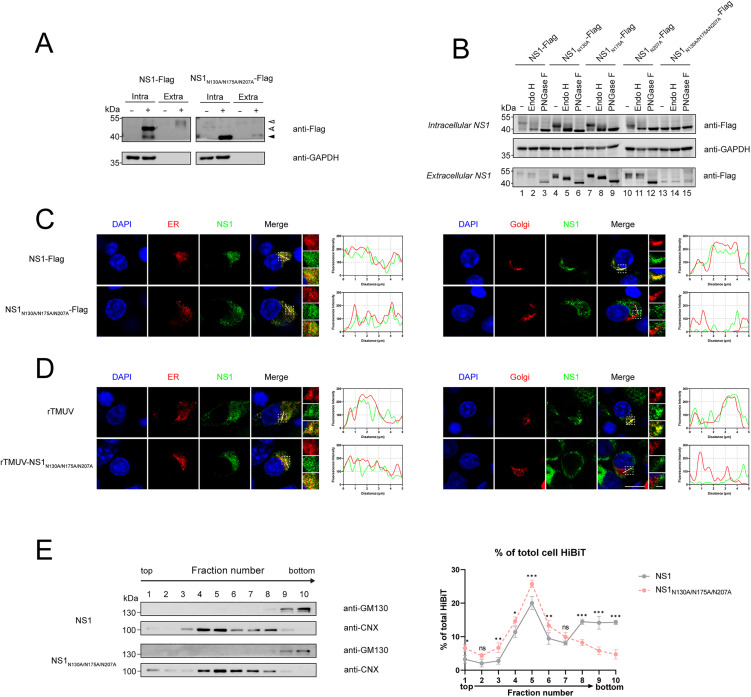
Deglycosylation inhibits the ER-Golgi transition of NS1. **(A)** Detection of intracellular and extracellular NS1. HEK-293T cells in 6-well plates were transfected with the indicated NS1-expression plasmids (2000 ng/well) for 48 **h.** NS1 in cell lysate (intracellular) and cell culture supernatant (extracellular) were subjected to western blot analysis using anti-Flag mAb (1:5000) or anti-GAPDH mAb (1:5000). Empty triangle represents the mature NS1, open white arrow corresponds to the immature NS1 and filled triangle represents the deglycosylated NS1. **(B)** Deglycosylation analysis of recombinant NS1. HEK-293T cells in 6-well plates were transfected with the indicated NS1-expression plasmids (2000 ng/well) for 48 **h.** Cell culture supernatant and cell lysate were subjected to Endo H or PNGase F treatment, followed by western blot analysis using anti-Flag mAb (1:5000) or anti-GAPDH mAb (1:5000). **(C and D)** Co-localization of NS1 with the ER and the Golgi apparatus in transfected or TMUV-infected cells. **(C)** BHK-21 cells in 12-well plates were co-transfected with the indicated NS1-expression plasmids (800 ng/well) and pDsRed2-ER or pDsRed2-Golgi (800 ng/well) for 36 **h. (D)** BHK-21 cells in 12-well plates were infected with rTMUV or rTMUV-NS1_N130A/N175A/N207A_ at 300 TCID_50_ for 12 h, then cells were transfected with pDsRed2-ER or pDsRed2-Golgi (1000 ng/well) for 36 **h.** The cells were incubated with mouse anti-Flag mAb or anti-NS1 mAb (clone 3D5), followed by staining with Alexa Fluor 488-conjugated anti-mouse IgG as the secondary antibody. The nuclei were stained with DAPI. The fluorescence signals were observed and imaged using confocal microscopy. The fluorescence intensity profile of NS1 (green) and the ER/Golgi apparatus (red) was measured along the line drawn by ImageJ software. Scale bar, 10 μm or 2 μm. **(E)** Detection of HiBiT activity derived from cell lysates by subcellular fractionation and normalized representation of the amount of NS1 in each fraction. BHK-21 cells in 100-mm culture dishes were transfected with NS1-HiBiT or NS1_N130A/N175A/N207A_-HiBiT for 48 **h.** The cellular contents were analyzed by fractionation on an iodixanol gradient. Ten fractions were collected from the top of the gradient, and the HiBiT activity of each fraction was measured. The Y-axis displays the percentage of HiBiT activity detected in each fraction. Means and SDs from three independent experiments are presented. *, P < 0.05; **, P < 0.01; ***, P < 0.001; ns, not significant. Each fraction was also evaluated by western blot analysis for the presence of CNX (ER marker) and GM130 (Golgi apparatus marker).

Glycosylation regulates protein folding in the ER lumen, ensuring that only properly folded glycoproteins are transported to the Golgi apparatus [64]. Therefore, we explored whether deglycosylation causes misfolded NS1 to be trapped in the ER by western blot. We found that intracellular and extracellular WT NS1 exhibit different electrophoretic mobilities (Fig 4A, open white arrow vs. empty triangle), whereas those of the NS1_N130A/N175A/N207A_ showed no differences (Fig 4A, filled triangle). Intracellular NS1 is sensitive to Endoglycosidase H (Endo H) treatment, consistent with its synthesis in the ER. However, during transport through the Golgi apparatus, it acquires partial Endo H resistance because of trimming of high mannose-type glycans to complex-type glycans [12]. We observed that the extracellular WT NS1 was partially Endo H-resistant (Fig 4B, lane 2) while the intracellular WT NS1 was mainly Endo H-sensitive (Fig 4B, lane 2). Moreover, the extracellular NS1_N207A_ remains Endo H-resistant, as it retains intact complex-type glycans (Fig 4B, lane 11). The mobilities of the NS1_N130A/N175A/N207A_ were almost identical to those of the WT NS1 treated with Endo H or Peptide N Glycosidase F (PNGase F) (Fig 4B, lane 2, 3 and 13). Moreover, intracellular and extracellular NS1_N130A/N175A/N207A_ exhibited unaltered mobility after Endo H or PNGase F digestion (Fig 4B, lane 13, 14 and 15), indicating that deglycosylated NS1 cannot undergo the secretory pathway from the ER to the Golgi apparatus. Immunofluorescence assay (IFA) was further preformed to assess the subcellular localization of NS1. We found that regardless of overexpression or viral infection, deglycosylation did not alter the co-localization of NS1 with the ER (Fig 4C and 4D, left panel) but impaired its co-localization with the Golgi apparatus (Fig 4C and 4D, right panel). To facilitate the quantification and detection of NS1, we fused a HiBiT tag to the C-terminus of NS1. Subcellular fractionation assays were performed to quantify NS1 by measuring HiBiT activity in each fraction. CNX and GM130 served as markers for the ER and the Golgi apparatus, respectively. We found that the ER was primarily localized in fraction 5–8, while the Golgi apparatus was mainly accumulated in fraction 9–10 (Fig 4E, left panel). For WT NS1, two HiBiT peaks were observed in fractions 5 and 8, reflecting NS1 trafficking through the secretory pathway from the ER to the Golgi apparatus; in contrast, deglycosylation resulted in a single HiBiT peak in fraction 5, with a higher percentage of NS1 in fraction 4–7, indicating that deglycosylated NS1 was arrested in the ER (Fig 4E, right panel). Taken together, these results indicate that NS1 glycosylation is required for its transport from the ER to the Golgi apparatus.

### Deglycosylation of NS1 inhibits the ER-to-Golgi trafficking of E proteins

**Fig 5:**
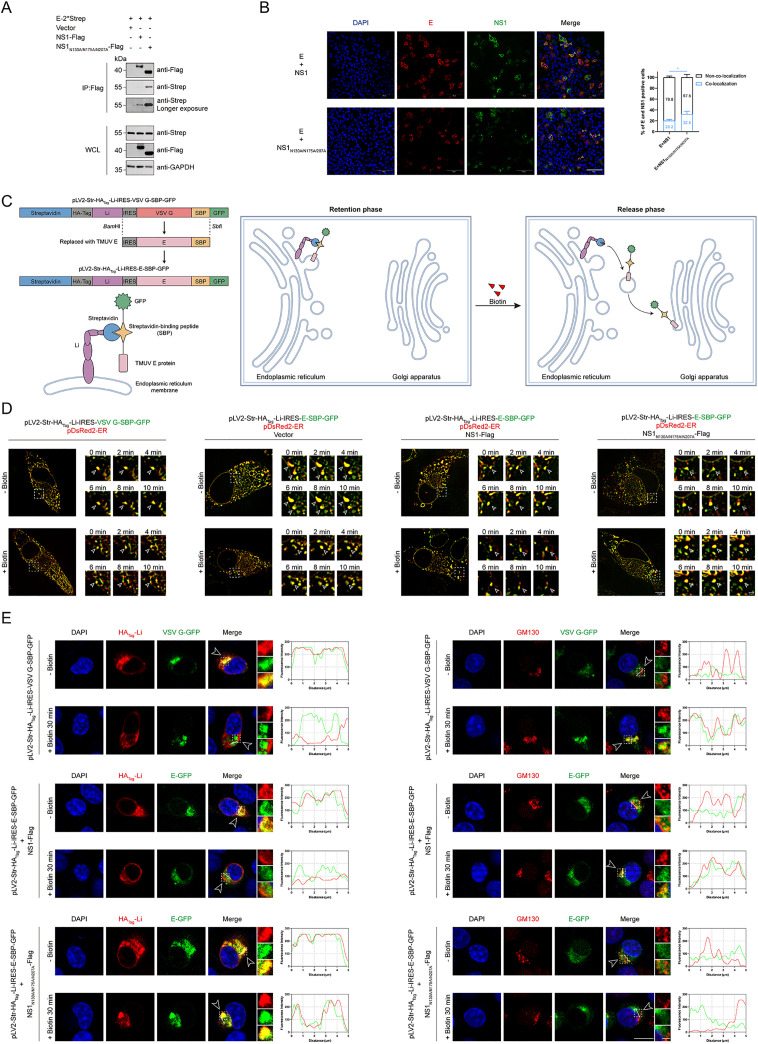
Deglycosylation of NS1 inhibits the ER-Golgi transition of E protein. (A) Detection the interaction between exogenous E protein and NS1. HEK-293T cells in 60-mm culture dishes were co-transfected with E-2*Strep (2000 ng/dish) and the indicated NS1-expression plasmids (2000 ng/dish) for 24 h. The cell lysate then was subjected to coimmunoprecipitation with anti-Flag mAb and western blot analysis. (B) Co-localization of NS1 and E protein. Hela cells in 12-well plates were co-transfected with E-2*Strep (800 ng/well) and the indicated NS1-expression plasmids (800 ng/well) for 36 h. Cells were incubated with mouse anti-Flag mAb and rabbit anti-Strep mAb, followed by staining with Alexa Fluor 488-conjugated anti-mouse IgG and Alexa Fluor 568-conjugated anti-rabbit IgG as the secondary antibodies. The nuclei were stained with DAPI. The fluorescence signals were observed and imaged using confocal microscopy. Cells showing co-colocalization are indicated by open white arrows. Scale bar, 100 μm. The percentage of cells showing co-colocalization was quantified using ImageJ software. (C) Schematic of genes coding for the hook (streptavidin-HA_Tag_-Li) and the reporter (VSV G/E-SBP-GFP), expressed under the same CMV promoter, separated by an internal ribosome entry site (IRES) (left panel); Schematic of the RUSH system (right panel). In the absence of biotin, SBP-tagged cargo is retained in the ER by the hook (ER-localized). After biotin addition, the reconstituted GFP-labeled cargo leaves the ER and translocate to the Golgi apparatus. Image was created from BioRender. (D) Timelapse imaging analysis of GFP-labeled cargos under the RUSH system. BHK-21 cells in 35-mm dishes were co-transfected with pLV2-Str-HA_Tag_-Li-IRES-E-SBP-GFP, pDsRed2-ER and indicated NS1-expression plasmids for 24 h, followed by addition of biotin (400 μM). The pLV2-Str-HA_Tag_-Li-IRES-VSV G-SBP-GFP was used as a positive control. Images of GFP and DsRed2 fluorescence are shown and elapsed times after addition of biotin are indicated at the top of each panel. Scale bar: 5 μm or 1 μm. (E) BHK-21 cells were co-transfected with pLV2-Str-HA_Tag_-Li-IRES-E-SBP-GFP and indicated NS1-expression plasmids for 24 h, followed by the addition of biotin (400 μM) to the culture medium. At 30 min after biotin addition, cells were fixed, permeabilized, and stained with anti-HA tag mab or anti-GM130 mab, followed by staining with Alexa Fluor 568-conjugated anti-rabbit IgG as the secondary antibodies. The nuclei were stained with DAPI. The fluorescence signals were observed and imaged using confocal microscopy. The fluorescence intensity profile of E/VSV G (green) and ER/Golgi apparatus (red) was measured along the line drawn by ImageJ software. Scale bar, 10 μm or 2 μm.

As orthoflavivirus E protein primarily participates in viral assembly, we investigated whether deglycosylation affects the interaction between NS1 and E protein using Co-IP and IFA. Interestingly, deglycosylation significantly enhanced the interaction between NS1 and the E protein (Fig 5A). Moreover, compared with WT NS1, a higher proportion of cells exhibited co-localization of the NS1_N130A/N175A/N207A_ mutant and E protein (Fig 5B, left panel, open white arrows). Consistently, the percentage of cells showing co-localization between the E protein and NS1_N130A/N175A/N207A_ was higher than that between the E protein and WT NS1 (Fig 5B, right panel). Given that NS1 deglycosylation trapps NS1 within the ER and enhances the NS1-E protein interaction, we speculate that this event may also impede the ER-to-Golgi trafficking of E proteins. The retention using selective hooks (RUSH) system utilizes the streptavidin-binding peptide (SBP), which binds streptavidin with high affinity; this binding interaction can be outcompeted by biotin, thereby enabling the synchronous release of viral proteins from the ER (anchored by Li) [49] (Fig 5C, left panel). This system enables visualization of the trafficking pathway of E proteins (Fig 5C, right panel). We used the representative vesicular stomatitis virus glycoprotein (VSV G), which is regarded as a model membrane protein, as a positive control. The results showed that in the absence of biotin addition, both VSV G and E proteins were anchored to the ER, with GFP and DsRed2 fluorescence co-localized; this co-localization persisted for at least 10 min (Fig 5D, upper panel). Following biotin addition, we observed that VSV G anchored to the ER could be released at 8 minutes (Fig 5D, bottom panel). As expected, WT-NS1 had no effect on the trafficking of E protein, as E-GFP dissociated from the ER at 8 min (Fig 5D, bottom panel). In contrast, deglycosylated NS1 disrupted E protein trafficking, as E protein remained colocalized with the ER (Fig 5D, bottom panel). The IFA results further demonstrated that in the absence of biotin, both VSV G and E proteins colocalized with the ER but not the Golgi apparatus (Fig 5E, left panel). Following biotin addition, most VSV G anchored to the ER could be released and reach the Golgi apparatus at 30 minutes (Fig 5E, upper panel). Consistently, we found that 30 min after biotin addition, a large number of E proteins were retained in the ER in cells transfected with NS1_N130A/N175A/N207A_ (Fig 5E, bottom panel), indicating that this mutant prevents the efficient trafficking of E proteins to the Golgi apparatus. Taken together, these results suggest that NS1 glycosylation regulates the transport of E proteins from the ER to the Golgi apparatus.

### NS1 deglycosylation induces degradation of NS1 and E through the host proteasome pathway

**Fig 6:**
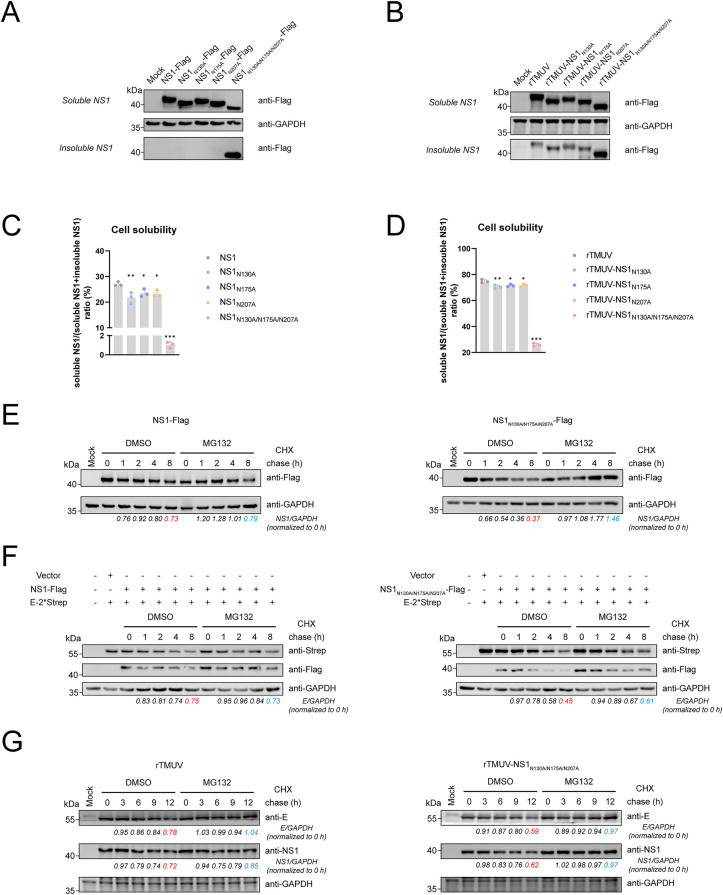
Deglycosylation of NS1 impairs its solubilization and induces rapid degradation of NS1-E by the host proteasomal pathway. **(A)** Western blot analysis of NS1 solubility in transfected cells. HEK-293T cells in 6-well plates were transfected with the indicated NS1-expression plasmids (2000 ng/well). At 48 h post-transfection, the cells were suspended in the RIPA buffer. Subsequently, the soluble and insoluble fractions were separated via centrifugation and subjected to western blot analysis using anti-Flag mAb (1:5000) or anti-GAPDH mAb (1:5000). **(B)** Western blot analysis of NS1 solubility in infected cells. BHK-21 cells in 6-well plates were infected with the rTMUV or various mutants at 3000 TCID_50_. At 48 h post infection, the cells were suspended in the RIPA buffer. Subsequently, the soluble and insoluble fractions were separated via centrifugation and subjected to western blot analysis using anti-NS1 mAb (clone 10F12) or anti-GAPDH mAb (1:5000). **(C)** HiBiT activity measurement analysis of NS1 solubility. HEK-293T cells in 6-well plates were transfected with the indicated NS1-HiBiT-expression plasmids (2000 ng/well). At 48 h post-transfection, the cells were suspended in the RIPA buffer. After centrifugation, HiBiT activities in the soluble and insoluble fractions were measured. The solubility of NS1 was calculated using the following formula: HiBiT value in the soluble fraction/total HiBiT value of the soluble and insoluble fractions. *, P < 0.05; **, P < 0.01; ***, P < 0.001; ns, not significant. **(D)** Indirect ELISA analysis of NS1 solubility in infected cells. NS1 levels in the soluble and insoluble fractions from (B) were quantified by indirect ELISA using anti-NS1 mAb (clone 8E6). **(E)** Stability of NS1. Cycloheximide (CHX) chase analysis was performed to evaluate the stability of NS1. HEK-293T cells in 6-well plates were transfected with the NS1-Flag or NS1_N130A/N175A/N207A_-Flag (2000 ng/well). After 24 h post-transfection, 50 μg/ml of CHX with DMSO or 10 μM MG132 were added to the cell culture. Samples were collected at the indicated time points and then were subjected to western blot analysis using anti-Flag mAb (1:5000) and anti-GAPDH mAb (1:5000). The intensities of the indicated protein bands were quantified by using ImageJ software. Data were shown as the fold-change of NS1/GAPDH. **(F)** Stability of E protein. CHX chase analysis was performed to evaluate the stability of E protein in NS1- or NS1_N130A/N175A/N207A_-transfected cells. HEK-293T cells in 6-well plates were co-transfected with the E-2*Strep (1000 ng/well) and NS1-Flag or NS1_N130A/N175A/N207A_-Flag (1000 ng/well). After 24 h post-transfection, 50 μg/ml of CHX with DMSO or 10 μM MG132 were added to the cell culture. Samples were collected at the indicated time points and then were subjected to western blot analysis using anti-Flag mAb (1:5000), anti-Strep mAb (1:3000) or anti-GAPDH mAb (1:5000). The intensities of the indicated protein bands were quantified by using ImageJ software. Data were shown as the fold-change of E/GAPDH. **(G)** Stability of infection-derived NS1 and E protein. BHK-21 cells in 6-well plates were infected with the rTMUV or various mutants at 3000 TCID_50_. After 36 h post infection, 50 μg/ml of CHX with DMSO or 10 μM MG132 were added to the cell culture. Samples were collected at the indicated time points and then were subjected to western blot analysis using anti-E pAb (1:3000), anti-NS1 mAb (clone 10F12) or anti-GAPDH mAb (1:5000). The intensities of the indicated protein bands were quantified by using ImageJ software. Data were shown as the fold-change of E/GAPDH and NS1/GAPDH.

We further measured the solubility of NS1. Transfected or TMUV-infected cells were lysed in RIPA buffer. Following centrifugation, the supernatant (soluble fraction) and pellet (insoluble fraction) were analyzed for NS1 levels by western blotting, HiBiT activity assay or indirect ELISA. Our results demonstrated that deglycosylated NS1 exhibited markedly reduced solubility under both overexpression (Fig 6A) and viral infection (Fig 6B) conditions. Consistently, compared with WT NS1, the NS1_N130A/N175A/N207A_ mutant displayed approximately ~25.2-fold and ~1.9-fold decreases in solubility under overexpression (Fig 6C) and viral infection (Fig 6D) conditions, respectively. Collectively, these results indicate that glycosylation plays a critical role in maintaining the solubility of NS1. Misfolded proteins in the ER lumen are ultimately transported to the cytoplasm, where they are degraded via the proteasomal pathway [65]. Given that NS1 is trapped in the ER and its solubility has decreased, we assessed the stability of WT NS1 and its mutants. Cycloheximide (CHX) chase analysis revealed that the NS1_N130A/N175A/N207A_ mutant exhibited a more rapid degradation rate than WT NS1, and the degradation of both was inhibited by the proteasomal inhibitor MG132 (Fig 6E). We further evaluated the effect of NS1 and its mutants on E protein stability and found that when co-expressed with NS1_N130A/N175A/N207A_ mutant rather than WT NS1, the E protein underwent more rapid degradation via the proteasomal pathway (Fig 6F). Consistently, we observed that both the infection-derived NS1_N130A/N175A/N207A_ and E protein underwent accelerated degradation during TMUV infection (Fig 6G). These findings indicate that NS1 deglycosylation reduces NS1 stability and leads to the degradation of both NS1 and E proteins through the host proteasomal pathway.

### NS1 deglycosylation regulates the progeny virion assemble in mutiple orthoflaviviruses

**Fig 7:**
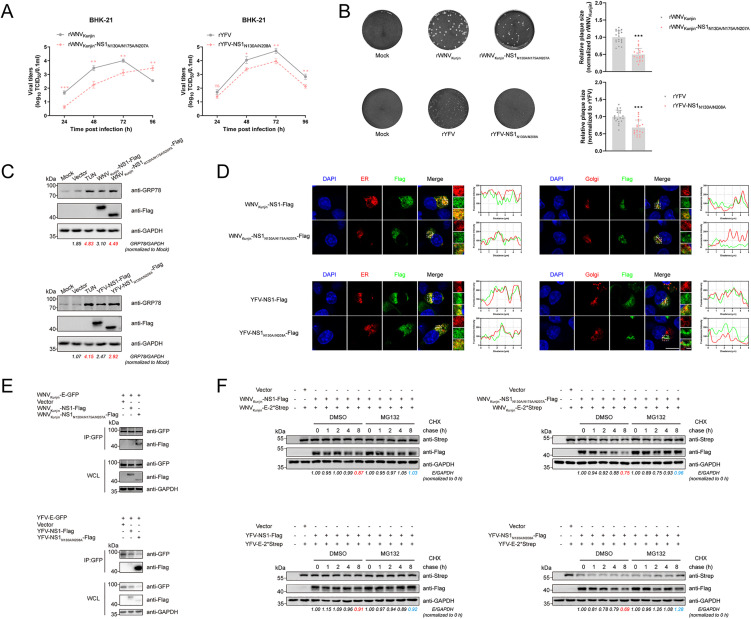
NS1 deglycosylation acts as a negative regulator for orthoflaviviruses. **(A)** Growth kinetics of rWNV_Kunjin_, rYFV, and their mutants in BHK-21 cells. BHK-21 cells in 12-well plates were infected with rWNV_Kunjin_, rYFV or their various mutants at 300 TCID_50_, and the culture supernatants were collected at the indicated time points. The viral titers were then determined by TCID_50_ assays. Means and SDs from three independent experiments are presented. *, P < 0.05; **, P < 0.01; ***, P < 0.001; ns, not significant. **(B)** Plaque morphology of rWNV_Kunjin_, rYFV and their mutants on BHK-21 cells. BHK-21 cells in 12-well plates were infected with 50 TCID_50_ of the indicated viruses and incubated in overlay medium supplemented with 2% FBS and 1% methyl cellulose. After 6 days, cells were fixed with 4% formaldehyde for 20 min and stained with 1% crystal violet for 1 min. Plaque sizes were measured via ImageJ software. **(C)** Effects of deglycosylated WNV_Kunjin_ or YFV NS1 on ER stress. BHK-21 cells in 12-well plates were transfected with the indicated NS1-expression plasmids (1000 ng/well). Cell lysate was harvested at 48 h post transfection and subjected to western blot analysis using anti-GRP78 pAb (1:3000) or anti-GAPDH mAb (1:5000). Cells treated with 10 mg/ml tunicamycin for 6 h were served as a positive control. Data were shown as the fold-change of GRP78/GAPDH. **(D)** Co-localization of WNV_Kunjin_ or YFV NS1 with the ER and the Golgi apparatus in transfected cells. BHK-21 cells in 12-well plates were co-transfected with pDsRed2-ER/pDsRed2-Golgi and the indicated WNV_Kunjin_-NS1- or YFV-NS1-expression plasmids for 36 **h.** Then cells were incubated with mouse anti-Flag mAb, followed by staining with Alexa Fluor 488-conjugated anti-mouse IgG as the secondary antibody. The nuclei were stained with DAPI. The fluorescence signals were observed and imaged using confocal microscopy. The fluorescence intensity profile of NS1 (green) and ER/Golgi apparatus (red) was measured along the line drawn by ImageJ software. Scale bar, 10 μm or 2 μm. **(E)** Detection the interaction between exogenous E proteins and NS1 of WNV_Kunjin_ or YFV. HEK-293T cells in 60-mm culture dishes were co-transfected with WNV_Kunjin_-E-GFP or YFV-E-GFP (2000 ng/dish) and the indicated NS1-expression plasmids (2000 ng/dish) for 24 h. The cell lysate then was subjected to coimmunoprecipitation with anti-GFP mAb and western blot analysis. **(F)** Stability of orthoflavivirus E protein. CHX chase analysis was performed to evaluate the stability of WNV_Kunjin_ or YFV E protein in transfected cells. HEK-293T cells in 6-well plates were co-transfected with the E-2*Strep (1000 ng/well) and indicated NS1-expression plasmids (1000 ng/well). After 24 h post-transfection, 50 μg/ml of CHX with DMSO or 10 μM MG132 were added to the cell culture. Samples were collected at the indicated time points and then were subjected to western blot analysis using anti-Flag mAb (1:5000), anti-Strep mAb (1:3000) or anti-GAPDH mAb (1:5000). The intensities of the indicated protein bands were quantified by using ImageJ software. Data were shown as the fold-change of E/GAPDH.

To explore whether NS1 deglycosylation exerts a regulatory effect on other orthoflaviviruses, we selected two viruses: WNV_Kunjin_, which has three N-glycosylation sites at N130, N175, and N207 like TMUV; and YFV, which has only two N-glycosylation sites at N130 and N208. Notably, rWNV_Kunjin_-NS1_N130A/N175A/N207A_ exhibited delayed viral proliferation. Consistent with observations for TMUV, rYFV-NS1_N130A/N208A_ showed impaired viral proliferation (Fig 7A). Both rWNV_Kunjin_-NS1_N130A/N175A/N207A_ and rYFV-NS1_N130A/N208A_ formed smaller plaque sizes (Fig 7B), confirming the role of NS1 deglycosylation in inhibiting orthoflavivirus proliferation *in vitro*. Western blot analysis demonstrated that NS1 deglycosylation of WNV_Kunjin_ or YFV also induces ER stress in transfected-cells (Fig 7C). IFA results further demonstrated that deglycosylated NS1 from WNV_Kunjin_ or YFV lost co-localization with the Golgi apparatus (Fig 7D). Co-IP results revealed that NS1 deglycosylation enhanced the NS1-E protein interaction for both WNV_Kunjin_ and YFV (Fig 7E). Finally, we consistently found that the E protein of WNV_Kunjin_ or YFV underwent rapid degradation via the proteasomal pathway when co-expressed with deglycosylated NS1 mutants rather than WT NS1 (Fig 7F). Taken together, these results indicate a universal phenomenon of orthoflaviviruses by which NS1 deglycosylation enhances the NS1-E interaction, which triggers the degradation of both NS1 and E via the proteasomal pathway, and thereby inhibits production of progeny viral particles.

## Discussion

Orthoflaviviruses are single-stranded positive-sense RNA arthropod-borne viruses. Many of these viruses are significant human pathogens—including dengue virus (DENV), Zika virus (ZIKV), yellow fever virus (YFV), West Nile virus (WNV), and Japanese encephalitis virus (JEV)—which pose a substantial threat to global public health. Orthoflavivirus nonstructural protein 1 (NS1) is one of the most multifunctional viral proteins: it is not only essential for viral replication [13–18] but also secreted from virus-infected cells as a hexamer glycoprotein. Glycosylation is an essential post-translational modification for the proper folding and function of glycoproteins, and plays a critical role in numerous cellular processes [58,66]. In eukaryotic cells, N-glycosylation stands as the most common form among other major types of glycosylation [67]. The mechanism of N-glycosylation is essential for orthoflavivirus proliferation *in vitro* [66]. For example, deglycosylation of DENV-2 NS1 at residues N130 or N207 exhibited a reduced viral proliferation in C6/36 cells and a smaller plaque size, respectively [39]. YFV NS1 lacking the first glycosylation site at N130 or both sites at N130 and N208 exhibits impaired viral proliferation *in vitro* [35]. The NS1_130–132QQA/175A/207A_ mutant of WNV showed a significant reduction in infectivity titer compared to the parental strain at all time points [60]. Here, we consistently found that the deglycosylation of TMUV NS1 reduces viral proliferation *in vitro*, and the phenotype is cell line independent (Fig 1A). Moreover, it was shown for YFV or WNV_Kunjin_ that deglycosylation of NS1 exhibited a delayed or impaired viral proliferation (Fig 7A), indicating the key role of NS1 N-glycosylation in orthoflavivirus proliferation.

During orthoflavivirus infection, the mechanism of N-glycosylation is also critical to the various biological functions of NS1, which in turn to involved in viral proliferation. Mutations at residues N130 or N207 of DENV NS1 do not affect its dimerization but compromise dimer stability [56], which is consistent with our results that deglycosylation does not affect the formation of NS1_N130A/N175A/N207A_ dimers in multiple cell types (Fig 2A and 2B) but impairs their thermal stability at 50°C (Fig 2D). Moreover, compared with NS1 derived from DENV-infected mammalian cells, NS1 derived from mosquito cells, which lacks the enzymes to generate complex-type glycans, was degraded more rapidly at 37°C [68]. In addition, compared to WT and N207Q mutant, N130Q and N130/N207Q mutants showed reduced secretion level of NS1 [59]. The secretion of NS1 was reduced when the processing of complex-type glycans was blocked by glycosylation inhibitors [21]. Interestingly, our results revealed that mutation of any single N‑glycosylation site on NS1 did not significantly impair its secretion efficiency (Fig 2E and 2G). This observation can be explained by two key reasons: (1) Orthoflavivirus NS1 must first form homodimers that further oligomerize into hexamers to support efficient secretion. However, our data demonstrated that neither single‑site glycosylation mutations nor the triple mutation disrupted dimerization of NS1 monomers (Fig 2A and 2B). Therefore, we reasonably speculate that N‑glycosylation maintains the conformational stability of NS1 by modulating the protein’s surface hydrophilicity and hydrophobicity, thereby facilitating oligomerization of NS1 dimers. In support of this hypothesis, previous studies reported that DENV NS1_N130Q/N207Q_, but not the single‑site mutant NS1_N207Q_, markedly reduces NS1 hexamer levels [59]. Combined with our findings, these data suggest that fully deglycosylated dimeric NS1 exhibits a severely impaired ability to further oligomerize into hexamers, failing to assemble into a mature, secretion-competent conformation, which ultimately results in a remarkable decrease in extracellular secretion. (2) From the perspective of viral evolution, the retention of multiple glycosylation sites on orthoflavivirus NS1 likely represents an adaptive evolutionary strategy. Mutation or loss of modification at a single glycosylation site does not fully abolish NS1 secretion, ensuring that the virus retains basic replication and transmission fitness under host selective pressure. Collectively, these results demonstrate that N-glycosylation of NS1 does not function through a single site alone; instead, multiple N-glycosylation sites act synergistically to ensure the proper secretion and functional stability of NS1. We consistently found that complete deglycosylation of NS1 resulted in a significant decrease in the level of NS1 secreted into the supernatant (Fig 2F), which may be attributed to two main factors: (1) The stability of the NS1 dimer is compromised owing to altered protein conformation, and misfolded NS1 dimers may lead to impaired secretion of functional hexamers. (2) The transport pathways of NS1 are disrupted, which in turn impairs the maturation and secretion of the protein. These results, combined with the observation that NS1 carrying only high-mannose glycans is secreted at low levels from DENV-infected mosquito cells while NS1 is efficiently secreted from DENV-infected mammalian cells [21], indicate that the efficient secretion of NS1 depends on the glycosylation status of the host cell, and particularly requires complex-type glycans. Since extracellular NS1 can antagonize complement activation via distinct pathways during orthoflavivirus infection [22–24], thus one reason for the impaired viral proliferation may be due to the low levels of NS1 in the cell culture supernatant could not facilitate viral immune escape. Notably, our findings further indicate that tunicamycin treatment markedly reduces both NS1 secretion and infectious virion production (Fig 3F and 3G). However, tunicamycin is not only an inducer of endoplasmic reticulum (ER) stress but also a broad-spectrum inhibitor of N-linked glycosylation in the ER. The viral E protein is a glycoprotein and its glycosylation is indispensable for its correct folding, intracellular trafficking, and subsequent viral assembly. As tunicamycin non-selectively blocks global N-glycan addition, this inhibitor cannot be used to discriminate the individual contributions of NS1 glycosylation from those of E protein glycosylation. Accordingly, the attenuated production of infectious virions upon tunicamycin treatment is most reasonably ascribed to the synergistic effects of impaired glycosylation of both NS1 and the E protein.

A previous study revealed that abrogation of NS1’ via the NS2A S9F mutation significantly enhances the growth and packaging efficiency of WNV replicons in WNV-C-DENV2-prM/E stable packaging cell lines, without impairing viral genome replication [69]. These findings confirmed that genomes that failed to generate NS1’ were packaged more efficiently than WT genomes, indicating a role for NS1’ in orchestrating viral assembly. Recently, studies have explored the novel function of orthoflavivirus NS1 in virion formation [43–45]. DENV NS1 was demonstrated to modulates infectious particle production via interaction with the viral structural E protein [43]. Moreover, it was demonstrated that JEV NS1_I273H_ failed to be secreted into extracellular environments and lost its capacity to facilitate the formation of infectious viral particles [45]. Similar to the N-glycosylation of NS1, that of the orthoflavivirus E protein is thought to play a crucial role in viral replication, propagation, neurovirulence, and neuroinvasiveness [70–72]. Interestingly, a recent study demonstrated that deglycosylation of JEV E protein reduces the production of infectious viral particles [73], however, its molecular determinants that are involved in virus assembly remains unknown. In this study, we found that deglycosylation of NS1 enhancd its interaction with the E protein but significantly impaired viral assembly, indicating a novel, conserved strategy employed by multiple orthoflaviviruses—including TMUV, WNV_Kunjin_, and YFV—whereby NS1 N-glycosylation is involved in the assembly of progeny virions via affecting the NS1-E interaction.

**Fig 8:**
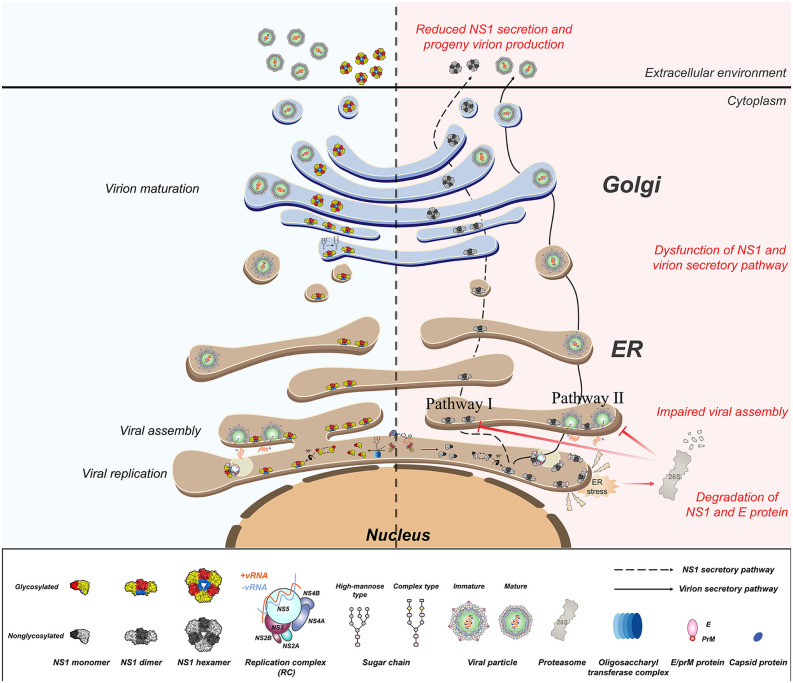
Model of the roles of NS1 N-glycosylation in NS1 secretion and viral particle formation. Deglycosylation of NS1 results in the retention of NS1 in the ER. On the one hand, this prevents further modification of NS1 in the Golgi apparatus, thereby disrupting the NS1 secretory pathway and reducing NS1 secretion (Pathway **Ⅰ)**. On the other hand, it enhanced the interaction between NS1 and E protein. Besides, retention of NS1 within the ER induces ER stress and subsequently causes rapid degradation of NS1 and E protein via the host proteasomal pathway. This impairs viral assembly, leading to dysfunction of the virion secretory pathway (Pathway **Ⅱ)**, and consequently reduces the production of infectious viral particles.

The maturation process of viral particles is closely associated with the glycosylation modification process of E protein, as these processes involve trafficking from the ER to the Golgi apparatus. It has shown that deglycosylation of the JEV E protein impaired the trafficking pathway of the E protein from the ER to the Golgi apparatus [73]. In this study, we successfully visualized and tracked the transport of E protein from the ER to the Golgi apparatus using the RUSH system (Fig 5D and 5E). This time-lapse imaging study confirmed that E protein release was inhibited by the deglycosylated NS1 secretion pathway. DENV NS1 was demonstrated to interact with the viral structural proteins, including C, prM and E[43]. Interestingly, our study further revealed that trafficking of the E protein from the ER to the Golgi apparatus is mediated by NS1 glycosylation, as deglycosylation disrupts the subcellular localization of NS1 and retains it within the ER. Owing to the enhanced NS1-E interaction, the E protein is also trapped in the ER. Additionally, we found that the solubility of deglycosylated NS1 is decreased. Misfolded proteins in the ER are ultimately transported to the cytoplasm, where they are degraded by the ubiquitin-proteasome system [65]. Consistently, we observed that both NS1 and E proteins are rapidly degraded via the host proteasomal pathway. Then, we extended our findings to YFV and WNV_Kunjin_, and observed similar results. Collectively, these results indicate that the trafficking pathway of NS1 is closely linked to that of virions through the NS1-E interaction. N-glycosylation of orthoflavivirus NS1 plays a crucial role in viral particle formation by maintaining viral assembly efficiency (Fig 8, left panel).

It is worth noting that a study revealed that glycosylation is critical for the proper folding, stability, and function of DENV NS1. The oligosaccharyltransferase (OST) complex mediates N-linked glycosylation of DENV NS1, and blocking this modification causes NS1 misfolding, retention in the ER, and impaired viral replication [74]. In addition, inhibition of NS1 glycosylation by the small-molecule glycosylation inhibitor celgosivir results in the retention of NS1 in the ER and blocked the transport of NS1 from the ER to the Golgi [75]. These data demonstrated that celgosivir inhibited DENV replication by impairing the folding and intracellular trafficking of DENV NS1, which is an essential part of its secretion process. Mechanistically, the calnexin (CNX) cycle serves as a core ER quality‑control system for glycoprotein folding, which depends on α-glucosidase-mediated trimming of N-linked glycans to mediate the interaction between viral glycoproteins and CNX/calreticulin chaperones. Inhibition of glycosylation by celgosivir weakens this chaperone-dependent quality-control process, preventing NS1 from acquiring its native conformation and resulting in NS1 misfolding. Misfolded NS1 fails to undergo normal intracellular trafficking and secretion, which directly impairs viral replication and reduces the production of infectious virions. Consistent with these observations in DENV models, our study further clarifies the mechanistic basis underlying this phenotype. Furthermore, excessive accumulation of misfolded NS1 in the ER lumen may induce ER overcrowding and indirectly disrupt processing of the viral E protein. As a key structural glycoprotein, the E protein matures within the ER and depends on intact ER folding machinery and sufficient chaperone resources for proper post-translational modification, folding, and assembly. Sequestration of ER chaperones by aggregated misfolded NS1, together with physical overcrowding of the ER compartment, impairs the normal processing and maturation of the E protein. Defective processing of the E protein further compromises viral assembly, representing an additional key mechanism underlying the reduced production of infectious virions. Collectively, these two interconnected mechanisms—impaired calnexin cycle-mediated quality control leading to NS1 misfolding, and ER overcrowding by misfolded NS1 disrupting E protein processing—together explain the impaired viral assembly caused by deglycosylated NS1, highlighting the indispensable role of NS1 glycosylation in maintaining proper virion assembly.

To the best of our knowledge, our studies are the first to elucidate the molecular mechanism that N-glycosylation of orthoflavivirus NS1 regulates viral assembly: the deglycosylation of NS1 disrupted its secretory pathway and significanty enhanced its interaction with E protein, as a consequence, both deglycosylated NS1 and E protein were arrested in the ER and could not be transported to the Golgi apparatus orderly, subsequently leading to a proteasome-mediated degradation, thereby disrupting the virus assembly, which further reduces the production of progeny viral particles (Fig 8, right panel). More importantly, the dependence of efficient progeny viral particle production on NS1 N-glycosylation is a conserved mechanism among multiple orthoflaviviruses. Taken together, our studies provide novel information for understanding the process by which orthoflavivirus NS1 regulates the assembly of progeny virions through its N-glycosylation. Targeting the N-glycosylation of NS1 serves as a promising platform for the development of broad-spectrum therapeutics against diverse orthoflaviviruses.
